# Considerations for fatty acids in standardized reference diet for parthenogenetic marbled crayfish *Procambarus virginalis* model organism

**DOI:** 10.1038/s41598-024-66268-7

**Published:** 2024-07-10

**Authors:** Koushik Das, Koushik Roy, Jan Mráz, Miloš Buřič, Antonín Kouba

**Affiliations:** Faculty of Fisheries and Protection of Waters, South Bohemian Research Center of Aquaculture and Biodiversity of Hydrocenoses, University of South Bohemia in České Budějovice, Zátiší 728/II, 389 25 Vodňany, Czech Republic

**Keywords:** Lipids, Proteins

## Abstract

Fatty acid accumulation was studied in the parthenogenetic all-female marbled crayfish *Procambarus virginalis* using six arbitrarily designed experimental feeds and related to individuals with glair glands (sexual maturity) after 100 days of ad libitum feeding at 21 °C, including gravid females from the wild as a reference. Fatty acids 16:0 and 18:1n-9 comprised 40% of the total amount of fatty acids and tended to up-concentrate in bodies. Shorter chain 14:0 depleted from feed to body. Across diets, there was a concomitant decrease in precursor fatty acid and increase in product fatty acid, such as reinforcements in monounsaturated fatty acid (18:1n-9), eicosanoid precursors 20:4n-6 (arachidonic acid, ARA) and 20:5n-3 (eicosapentaenoic acid, EPA) *in-vivo,* but not 22:6n-3 (docosahexaenoic acid, DHA) except when deficient in CHI or CHI + SPI diets. Saturation kinetics modeling (R^2^ 0.7–0.9, *p* < *0.05*) showed that when the ARA share is ~ 1%, the EPA share is ~ 8%, and the DHA share is ~ 2% in the food lipids, the accumulation of fatty acids in body lipids levels off. The lowest DHA in the CHI (0% glair glands) or CHI + SPI (0–3.9% glair glands) diets, and the lowest ARA in SER (0% glair glands) or SER + SPI (0–3% glair glands) diets, were synchronous with negligible sexual maturity despite a wide range of observed specific growth rates (2.77–3.60% per day), body size (0.44–0.84 g), ≤ 5% crude lipid and 40–46% crude protein feed. The FISH and SHRIMP diets (56% protein, 11–14% lipid) with the highest ARA, EPA, and DHA together seem to be the most conducive diets for sexual maturity (up to 20% of individuals with glair glands). We propose a fatty acid profile mimicking the FISH or SHRIMP diets as a starting point for designing the lipid content required in the marbled crayfish standardized reference diet.

## Introduction

When rearing animals in the laboratory for experimental needs, diet is a key factor that influences growth and reproduction. Unwittingly, diet may affect responses during experiments and cause variability in similar types of experiments undertaken in different laboratories^1^. Therefore, feed is a nutritional variable that could alter the physiology and bioenergetics of laboratory model organisms. Given that rearing conditions must be standardized, a standard feeding and nutritional regime is also part of a standardized rearing practice. Periodically, they are revised and agreed upon by international bodies that define and validate recommended scientific practices for laboratory model organism use in science (e.g., FELASA – Federation of European Laboratory Animal Science Associations). Therefore, standardized reference diets (SRD) for a given animal model and for all laboratories, which is part of a fixed rearing protocol, need to be established. A detailed prologue tackling this subject has recently been published as part of a zebrafish SRD-related review that discusses the evolution of the SRD concept in the rodent models used in the study of human medicine^1,2^. Thus, developing open-access recipes of SRD for model laboratory animals should be a priority^3^. However, most emerging laboratory models are nutritionally poorly known and not well understood. Therefore, it would take years of nutritional research to establish SRDs, above all if the species in question are not of commercial importance (e.g. not involved in aquaculture) and seldom studied by aquatic animal nutritionists. In such a situation, to fast-track SRD development, background work accelerating the process is needed. For example, a previous study on aging in a model vertebrate organism, the turquoise killifish *Nothobranchius furzeri*, showed that both its growth and reproductive potential are highly manipulable depending on the diet used in the laboratory^4^. In our previous study^5^, we explored how macronutrients in feed, energy, and amino acids affect somatic growth in marbled crayfish. Whereas, in this current study, we focus on the suitability of certain dietary fatty acid profiles using the appearance of the glair glands as a sign of maturation.

The marbled crayfish *Procambarus virginalis* has emerged as a good biological model for crustaceans^6,7^. It is a parthenogenetic freshwater species of recent evolutionary origin, first detected in the German pet trade with aquatic organisms. Following introductions to the wild, it has invaded diverse habitats, especially in Europe and Madagascar^8,9^. It was listed by the European Union as an invasive species of concern, threatening native crayfish populations and local ecosystems. It is known for establishing prolific, all-female monoclonal populations with good tolerance of environmental stress^6,10^. A common approach in laboratories is to feed these crayfish on diverse plant and animal feeds such as *Artemia* nauplii when early juveniles and/or on defrosted chironomid larvae with or without grated carrots when older^11–14^. In terms of artificial dry feed, pet food for aquarium (ornamental) fish such as Sera Ganugreen and Tetra Wafer mix are indeed commonly used^11,15–18^. Ornamental fish feed recipes are based on extrapolations of nutrient requirements and practices derived from food fish under intensive culture conditions aimed at maximum growth in a short time. Such nutritional requirements might be unsuitable for fish kept in public and home aquaria. They are also aimed at satisfying multiple species kept together in aquaria. As such, ornamental fish feeds are not intended to ensure optimum growth and physiology or be species-specific^19^. These traditionally used ornamental fish feeds are often deficient in certain nutrients, so growth can be slower^4,5^, causing micronutrient deficiencies^20^ and generational turnover may take longer. Although efforts have been made to understand preferences for amino acids in marbled crayfish^16^ (see our previous study^5^), research is yet to explore the preferences for fatty acids that affect the speed at which maturation and accompanied glair gland formation appear^21^.

Lipids are macronutrients that are essential for forming membranes and producing energy, and influence many physiological processes^22–24^. They are also involved in cell composition, the absorption and transport of fat-soluble vitamins, and the supply of essential fatty acids^25,26^. Some fatty acids including arachidonic acid (ARA, 20:4n-6), eicosapentaenoic acid (EPA, 20:5n-3), and docosahexaenoic acid (DHA, 22:6n-3) are implicated in the control of crustacean molting^27,28^. Dietary lipid sources have an impact on growth and gonad maturation in female crayfish, and also enhance vitellogenesis and gonad development^24,29–32^. Additionally, lipids play a crucial role in the production of embryonic tissue and serve as a vital source of organic metabolic energy for developing eggs^27,33^. Low lipid levels in broodstock diets diminish egg quality and lipid reserves stored in the hepatopancreas are insufficient to fulfill the requirements of the ovaries. Thus, diets must contain an adequate supply of lipids^33,34^. It is generally recognized that dietary long-chain (> C20) polyunsaturated fatty acids (LC-PUFAs) have implications on broodstock development and gamete quality^35^.

To explore the correlation between the dietary fatty acid supply and their accumulation in or disappearances from whole-body homogenates, this study implemented the formulation of six arbitrarily random fatty acid diets. Further in the study, we evaluated the reproductive potential of cultured stocks after 100 days of ad libitum feeding at 21 °C under different diets.

## Results

### Conduciveness of glair glands formation

The FISH (10.9% crude lipid) and SHRIMP (13.5% crude lipid) diets at iso-protein level (56% crude protein) led to the appearance of glair glands. One exceptional individual under the SHRIMP diet (Supplementary information Fig. S1) even became gravid; however, this group was the one with the highest rate of observed cannibalism (thus, in the SHRIMP diet group there was a greater supply of FAs – which could not be quantified – than in the pellet-origin). However, the second highest rate of cannibalism was in the SER group (Supplementary information Fig. S2), which was not sufficient to develop glair glands. This indicates that pellet composition plays a role in glair glands formation. The appearance of replicate-wise glair glands can be found in the Supplementary ﻿information (Table S6). Across dietary treatments with ≤ 5% crude lipids and 40–46% crude protein, there was none or negligible glair glands formation within a wide range of specific growth rates (2.77–3.60% per day) and body sizes (0.44–0.84 g) (Table 1). A detailed fatty acid profile of the experimental diets can be found in Table 2. The lowest DHA share in the CHI (0% glair glands) or CHI + SPI (0–3.9% glair glands) lipid diet, and lowest ARA share in SER (0% glair glands) or SER + SPI lipid diet (0–3% glair glands) were synchronous with the negligible appearance of glair glands. The FISH and SHRIMP diets with the highest ARA + EPA + DHA content – that also fulfilled individual thresholds of ARA, EPA, and DHA based on the saturation kinetics models given below – exhibited the most glair glands development (Table 1).

**Table 1:** Lipid content and target fatty acids composition of experimental diets and animals in terms of growth, sexual maturity, and mortality.

Diet (descending order of fattiness)	Feed lipid (% of pellet dry matter)	Body lipid (% of wet biomass)	Maturity (% crayfish with glair glands)	ARA + EPA + DHA (g 100 g^−1^)		Median^#^ SGR (%/ day)	Median^#^ final BW (g)	Mortality/ cannibalism%
				Feed (dry)*	Body (wet)*			
SHRIMP	13.49 ± 0.19	1.19 ± 0.08^b,c^	5.60 ± 4.00^a,b^	2.95 ± 0.03	0.22 ± 0.01^a^	3.46^a^	0.73^a^	50 ± 4.1^a^
FISH	10.91 ± 0.21	0.77 ± 0.02^a^	17.60 ± 4.10^b^	1.91 ± 0.05	0.15 ± 0.002^b^	4.08^b^	1.16^b^	30 ± 7.1^b^
SER	5.33 ± 0.02	0.90 ± 0.04^a^	0.00^a^	0.68 ± 0.001 (ARA deficient)	0.14 ± 0.004^b^	2.77^a^	0.44^a^	43.3 ± 6.2^c^
CHI	5.04 ± 0.02	1.27 ± 0.004^b^	0.00^a^	0.66 ± 0.001 (DHA deficient)	0.22 ± 0.006^a^	3.25^a^	0.53^a^	8.3 ± 2.4^b^
SER + SPI	3.94 ± 0.08	0.86 ± 0.10^a^	< 3.0^a^	0.48 ± 0.007 (ARA deficient)	0.15 ± 0.022^b^	3.60^a^	0.72^a^	30.0 ± 14.1^b^
CHI + SPI	3.28 ± 0.01	0.76 ± 0.07^a^	< 3.92^a^	0.33 ± 0.001 (DHA deficient)	0.15 ± 0.011^b^	3.45^a^	0.84^a^	18.3 ± 6.2^b^
Wild (mature)	–	0.82	100	–	0.15	–	–	–

*Feed is 91–93% dry pellet. The body is wet weight with approximately 25% dry matter.

^#^For mean, standard deviation, and range, see Das et al.^5^.

SHRIMP = Shrimp feed; FISH = Fish feed; SER = Sera Granugreen feed; CHI = Chironomids; SER + SPI = Sera Granugreen + Spirulina; CHI + SPI = Chironomids + Spirulina. ARA = Arachidonic acid; EPA = Eicosapentaenoic acid; DHA = Docosahexaenoic acid.

All feed had consistent dry matter values (91–93%).

A specific growth rate (SGR) is expressed as a % increment in average body weight (BW) per day.

Alphabetical superscripts refer to statistically significant differences between tested groups (*p* < 0.05).

**Table 2:**
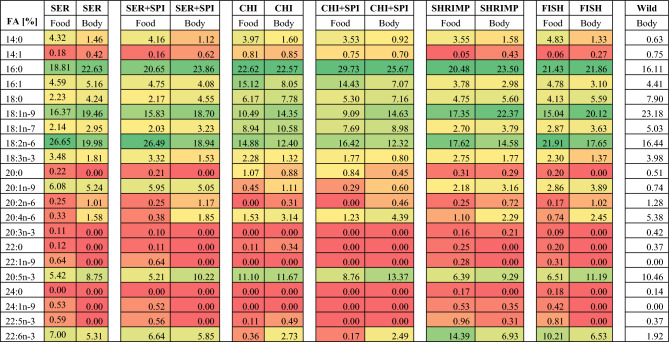
Fatty acids composition in experimental feed and crayfish compared to a wild control.

SER = Sera Granugreen feed; SER + SPI = Sera Granugreen + Spirulina; CHI = Chironomids; CHI + SPI = Chironomids + Spirulina; SHRIMP = Shrimp feed; FISH = Fish feed; FA = Fatty acids.

Values are percentage (%) of total lipid or fatty acids (total = 100%).

*Conditional formatting of the table*: low value = red to orangish color, medium value = yellow color, and high value = greenish to green color.

### Pattern of fatty acid use (feed-to-whole-body lipids)

By comparing the wild control (mature) and experimental groups, the saturated fatty acids (SFA) 16:0 (palmitic acid) and monounsaturated fatty acids (MUFA) 18:1n-9 (oleic acid) were found to represent ~ 40% of all fatty acids in the crayfish. From feed to body mass, precursor SFA 16:0, intermediary SFA 18:0, and product MUFA whole-body lipid 18:1n-9 combined showed a general tendency to up-concentrate (Table 2), while the shorter chain SFA 14:0 was depleted during feed to body mass conversion in all groups, irrespective of diets (Table 2).

**Figure 1:**
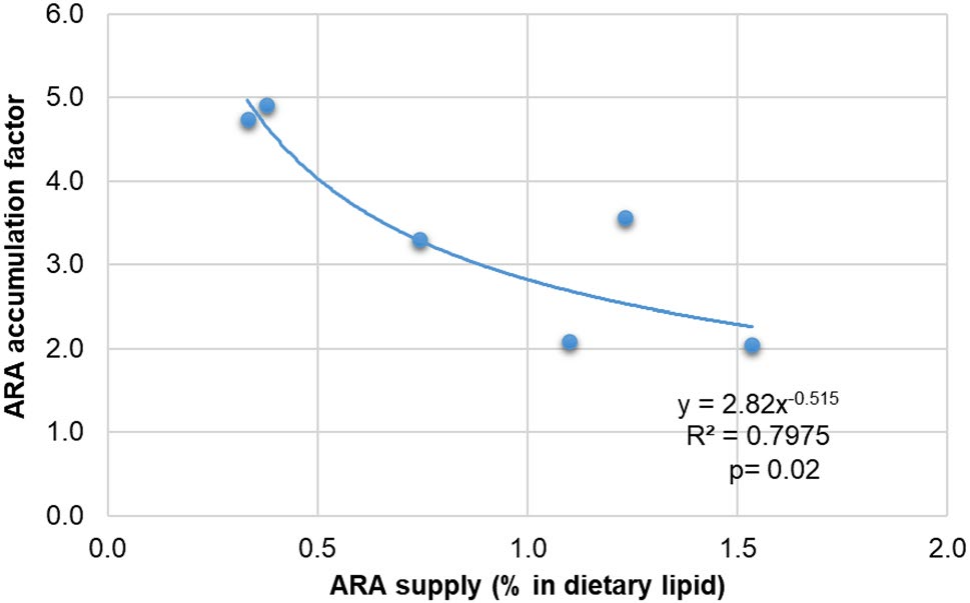
A saturation kinetics model for marbled crayfish for the physiological requirement of arachidonic acid (ARA), a possible essential fatty acid for the development of a standardized reference diet.

In all groups (Table 2), there was a concomitant decrease in 18:2n-6 from feed to body mass and a sequential increase in 20:2n-6 (an elongated product), as well as in 20:4n-6 (a desaturated product) from feed to body mass. This pattern implies that there was a reinforcement tendency towards ARA (20:4n-6). ARA levels in the wild sample seem to be much higher than in captive animals (Table 2). We also used a power analysis to model the dietary share of ARA in lipids in feed and the response in the ARA share in crayfish whole-body lipids. A statistically significant saturation kinetics model was evident, with 80% variability explained by the ARA share in feed and whole-body lipids (Fig. 1; R^2^ 0.80, *p* < *0.05*). When the ARA share is > 1% of lipids in pellets, its accumulation in body lipids levels off or becomes saturated (Fig. 1).

**Figure 2:**
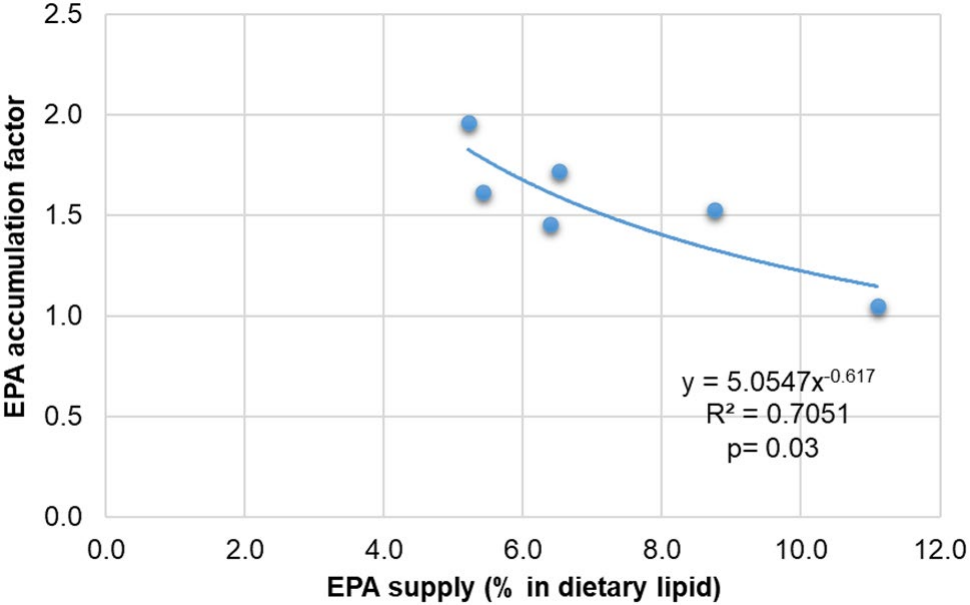
A saturation kinetics model for marbled crayfish for the physiological requirement of eicosapentaenoic acid (EPA), a possible essential fatty acid for the development of a standardized reference diet.

In all groups (Table 2), there was a concomitant decrease in 18:3n-3 from feed to body mass and an increase in 20:5n-3 (an elongated and desaturated product) from feed to body mass. This implies that there was a reinforcement tendency towards EPA (20:5n-3). Using a similar power analysis, a statistically significant saturation kinetics model was generated for EPA that explained 70% of variability between the EPA share in feed and the whole-body lipids (Fig. 2; R^2^ 0.70, *p* < *0.05*). When the EPA share is ~ 8% in feed lipids, the accumulation of EPA in body lipids slows down.

**Figure 3:**
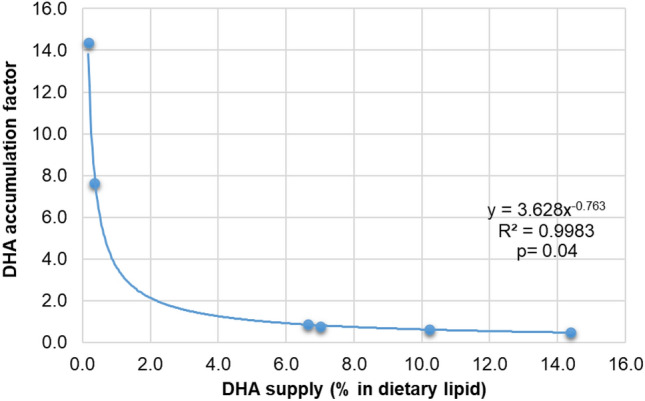
A saturation kinetics model for marbled crayfish for the physiological requirement of docosahexaenoic acid (DHA).

DHA (22:6n-3) was not reinforced in any of the diets except CHI and CHI + SPI, where DHA was lowest, less than 0.4% of lipids (Table 2). DHA comprised ~ 2% of the whole-body lipids in the wild control (Table 2). The statistically significant saturation kinetics model explained almost complete variability between the DHA share in the feed and the whole-body lipids (Fig. 3; R^2^ 0.99, *p* < *0.05*). The model indicates that DHA accumulation peaks when DHA is less than 2% of lipids in feed. Once this level is reached, the DHA accumulation levels off or becomes saturated.

## Discussion

Our previous study indicated that marbled crayfish prefer more of a ketogenic type of diet, with carnivorous-like metabolism having a higher preference for protein and certain amino acids^5^. Excess of dietary lipids in crustaceans has shown to inhibit growth and/or impair survival, as reviewed by D`Abramo^36^. The crayfish are lean in general, with whole-body fat content narrowly ranging 0.8–1.3% of wet weight only, irrespective of dietary fat content 3.3–13.5% (present study). It seems that overall fat reserve in the body is of lesser priority, but more priority is the reserve of specific fatty acids within the body lipids (Table 2). Hence, the dietary fatty acids profile seems important. Both ARA and EPA are eicosanoid precursor fatty acids^37^ to produce pro-inflammatory series-2 prostaglandins (PGE_2_) and reduced pro-inflammatory/ anti-inflammatory series-3 prostaglandins (PGE_3_), respectively. Of which, ARA-derived PGE_2_ is a high bioactivity eicosanoid^23^. In invertebrates (crustaceans), the cyclooxygenase enzyme responsible for PGE_2_ synthesis from ARA is present^38^. Eicosanoids, mainly prostaglandins, are needed for inflammatory responses and sexual steroidogenesis (hormones) needed for ovarian maturation, ovulation, expulsion of eggs out of gonopore, and signaling molecules for post-ovulatory ripening of eggs^39^. In procambarid crayfish, the conversion of ARA to PGE_2_ during the final stages of vitellogenesis has been previously observed^40^. The stimulatory effect of PGE_2_ is so high that, for instance, one-third of ‘virgin’ females (i.e. without any male intervention) showed egg-laying behavior in a non-parthenogenetic lepidopteran insect^41^. We did not measure prostaglandin in our samples, but postulate that an abundant supply of eicosanoid precursor fatty acids through SRD in parthenogenetic marbled crayfish may be of importance to aid prostaglandin synthesis, attainment of sexual maturity, and lower generational time of lab stocks including viable offsprings. Manipulating eicosanoids biosynthesis by SRD may be desirable for laboratory managers; for example, if breeding stocks are needed earlier or later. Or in cases where inflammatory response parameters (immunity/ stress related) are being recorded in some eco-toxicity studies, it may require certain level of endogenous eicosanoid pre-cursor fatty acid reserves like wild crayfish (*i.e.,* high 20:4n-6 and 20:5n-3 in Table 1, wild body), who have possibly high eicosanoid biosynthesis potential in nature. These mechanisms merit deeper investigation.

Our results suggest feeding an ARA and EPA-deficient diet may potentially slow down sexual maturation, as the crayfish must endogenously fortify them first (e.g., ARA/ EPA saturation kinetics models) and then achieve a threshold eicosanoids balance for egg laying. We observed possible endogenous fortifications of both ARA (from 18:2n-6) and EPA (from 18:3n-3) occurring in all diets, based on their change in values from food to body (Table 2). Recently, a complete Δ6 desaturase-like gene was characterized in the hepatopancreas of the redclaw crayfish *Cherax quadricarinatus*^42^, including elongase-coding ELOVL genes^43^. A combination of these two genes is attributed to 20-chain carbon PUFA synthesis such as ARA or EPA^44,45^, the precursors of prostaglandin synthesis^23^. Diets CHI and CHI + SPI were deficient in DHA but abundant in EPA; under these diets, the DHA in the crayfish body accumulated abundantly. The Δ6 desaturase can play a role in DHA synthesis (when needed) via the indirect Sprecher pathway^45^. Animals that had to endogenously fortify DHA in the CHI and CHI + SPI diets had the lowest rates of cannibalism. Aquatic predators have a higher circulation of ARA in blood derived from digested membranes (phospholipids) originating from animal prey^46^. The CHI and CHI + SPI diets had the highest share of ARA in the dietary lipids and also had the lowest cannibalism rates. However, survival rates are closely related to growth performance and the fastest-growing species tend to have higher survival rates (as discussed by Kouba et al*.*^15^).

Nonetheless, DHA is important for structural integrity, embryo development, and hatching success in fish eggs^47^. Despite different sources of lipids, i.e. originating from either artificial feed or natural food, DHA levels in eggs seem to be conserved, which cannot be said for EPA or ARA levels^48^. Marbled crayfish juveniles reach 30 mg some two weeks after the onset of exogenous feeding^15^. Adding this time to the age of the final stocks (100 days post-exogenous feeding) suggests that our crayfish first matured at around < 120 days post-hatching. Ripe eggs were eventually ovulated under the SHRIMP diet with the highest DHA. Such early maturation is in line with previous research by Kouba et al*.*^15^, who attained the first ovulating female after 14 weeks of culture at 22 °C, thereby suggesting that the earliest age of maturity is at ~ 110 days. The maturation process is temperature-dependent too, as shown by Seitz et al*.*^49^, although we feel it is also important to underline the importance of an optimized diet, rich in certain fatty acids (this study) or amino acids^5^.

Compared to SFA, the mitochondrial β-oxidation of MUFA and PUFA requires the participation of an extra set of three auxiliary enzymes. Thus, shorter-length SFAs are assimilated faster than lengthier or more complex ones^50^. In our study on the fatty acids profile, 14:0 was the shortest and simplest and was readily depleted during food-to-body mass conversion, whereas 16:0 → 18:0 → 18:1n-9 (part of SFA to MUFA biosynthesis) increased. Wu et al.^42^ recently characterized a Δ9 fatty acid desaturase-like gene from redclaw crayfish that performs MUFA synthesis^51^. To date, the metabolic use of fatty acids in crayfish is thought to follow the concept of fatty acid allostasis rather than homeostasis. According to the allostasis concept, the fatty acid profiles of crayfish reflect what they eat^50^. Our saturation kinetics model of DHA supports the concept of homeostasis. The ARA, EPA, or MUFA accumulation patterns from feed to body mass in all diets (including the depletion pattern of their precursors from food to body mass), hint at the ability of marbled crayfish to potentially upgrade some fatty acids in vivo. Future SRD could take these into account and also validate fatty acid bioconversions. One of the missing aspects of this study was an analysis of cholesterol, which is considered essential in crustaceans^52^. Therefore, the SRD_lipid_ for marbled crayfish should also take into account dietary cholesterol.

Our study had certain limitations. The varied macronutrient composition of our experimental diets may affect the interpretation of the findings, although the present study reports trends in vivo from an arbitrary selection of diets that could be common to all. The experiment could not be continued beyond 100 days, as possible hatching of eggs could alter headcounts (*i.e.,* inter-group comparison of survivability/ mortality compromised) and induce cannibalism with either females, eggs, or juveniles being food items rich in protein and phospholipids (*i.e.,* undermining inter-group comparison of the feeding value of pellets). Nonetheless, our observed trends could help decide on an appropriate dietary fatty acid composition for the parthenogenetic crayfish model, which could affect reproduction (e.g. using certain FA-deleted diets to delay maturation or FA-fortified diets to advance it) and thus be useful for laboratory managers. Iso-protein and optionally iso-calorific semi-purified research diets using different blends of oil could be used to calculate an optimum SRD fatty acid profile for marbled crayfish. The present study, in tandem with Das et al*.*^5^, could provide the needed baseline information for formulating hypotheses or controls for this type of experiment. The managerial implication of this study is to help better understand the lipid and fatty acid requirements in future marbled crayfish SRD.

## Methods

### Design and preparation of experimental feed

A detailed description of the experimental feed formulation can be found in Das et al*.*^5^, which elaborates on protein and amino acids requirements in laboratory model species, the marbled crayfish. A summary is provided here. Three protein levels were formed with amino acid manipulations nested in each level to comply with the ideal protein concept for wild-caught marbled crayfish bodies. Fatty acid manipulations nested under each protein level were randomized. The first level (~ 40% crude protein; ~ 11.9 kcal gross energy g^−1^ crude protein) contained Sera Granugreen (SER) and Sera Granugreen fortified with spirulina (SER + SPI). The second level (~ 46% crude protein; ~ 8.7 kcal gross energy g^−1^ crude protein) contained lyophilized chironomid larvae (CHI) and chironomid larvae fortified with spirulina (CHI + SPI). Finally, the third level (~ 56% crude protein; ~ 9.3 kcal gross energy g^−1^ crude protein) contained two commercially standardized larval diets, one for carnivorous fish (Skretting Perla larva 0.3–0.5 mm; hereafter FISH) and the other for shrimp post-larvae (Skretting Shrimp feed PL #3, 300–550 mm; hereafter SHRIMP). The lipid content and fatty acids profile of the experimental diets are shown in Tables 1 and 2, respectively. The composition of each diet is given in Tables S8–S13 in the Supplementary ﻿information. The manufacturing of the experimental pellets using the cold pelletization approach is described in detail in Das et al*.*^5^ and follows previous research by Žák et al*.*^4^. The proximate composition, amino acids, calcium, and phosphorus were analyzed in a third-party accredited laboratory (AGROLA, spol. s.r.o., Czech Republic https://agrola.cz/laborator/) using ISO/EU certified protocols. The methods included dry matter (method: ČSN ISO 11,465), ash (ČSN ISO 11,465), phosphorus (ČSN EN ISO 11,885), lipids (ČSN 46 7092–7), fiber (ČSN ISO 6541), protein (ČSN EN 16,634–1), and calcium (ČSN EN ISO 11,885). The nitrogen-free extract (NFE) was calculated as NFE = dry matter—(protein + lipid + fiber + ash)^5^.

### Crayfish keeping and husbandry

Wild-caught individual marbled crayfish were collected from Prostřední pond in Prague, Czech Republic. They were immediately placed on dry ice in an ice box and then transported to the laboratory for biochemical analysis. The juveniles (stocking material for experiments) were sourced from our laboratory stock at the Laboratory of Freshwater Ecosystems, FFPW, USB, Vodňany^53^. In total, 360 juvenile marbled crayfish with a mean weight of 30 ± 0.1 mg were used as experimental animals. This weight approximately corresponds to animals after two weeks of feeding under laboratory conditions (i.e. after the onset of exogenous nutrition in the third developmental stage)^14^. Stocking involved 20 individuals per aquaria; six experimental feeding groups were tested in triplicate. During stocking, size differences between tanks were tested and fixed at *p* > 0.05 across all tanks. The experiment was conducted for 100 days, under ad libitum feeding at an optimum feeding temperature (21 °C) in a series of indoor glass aquaria (54 × 36 × 30 cm, volume 46 L) fed by a water recirculating system^15^. Altogether, 18 aquariums were kept climatically stable (21 ± 1 °C) with an artificially maintained photo regime (12L:12D). A brick (28.5 × 13.5 × 6.5 cm) with 39 cross holes (26 and 13 holes with profiles of 1 × 3 cm and 1 × 1 cm, respectively) was placed in each aquarium to provide shelter^15^. Additionally, blocks of joined polypropylene tubes, each of five tubes (length 10 cm, inner diameter 35 mm), were placed in each aquarium as larger additional shelters for growing animals^15,54^.

Marbled crayfish juveniles were hand-fed ad libitum twice per day (~ 6% of tank biomass per day; at 08:00 and 15:00) with the abovementioned diets. Feeding was done to ensure complete satiation and minimize cannibalism. The feed was usually spread over the whole of the tanks to ensure equal access for all animals and avoid feed-induced aggression. Uneaten feed, feces, and other waste materials were siphoned out manually each morning. Dissolved oxygen (8.9 ± 0.5 mg L^−1^), pH (7.3 ± 0.3), and temperature (21 ± 1 °C) were measured daily using Oxi 3205 and pH 720 m (WTW GmbH, Weilheim, Germany). The body weight of marbled crayfish from each aquarium was measured every 14 days with an electronic balance (lowest sensitivity 1 mg) and the number of survivors was counted. Body weight measurements were taken before feeding. At the end of the experiment (100 days), the animals were starved for one day, their final body weight was taken (Supplementary ﻿information Table S6) and the presence of glair glands and attached eggs were visually noticed^21^. For categorization of crayfish females reproduction, see Supplementary ﻿information Fig. S3. The experiment could not be continued beyond 100 days, as elaborated at the end of discussions.

### Lipid and fatty acids analyses

The preparation of finely pulverized crayfish whole-body homogenate (including exoskeleton, antennae, and appendages) was done in accordance with established protocol^53^. The lipid extraction followed the Folch method^55^ with slight modifications^56^. The quantification of the lipid content was done gravimetrically using a pre-established protocol in the laboratory^57,58^. In brief, approximately 1 g of crayfish whole body homogenate or homogenized feed pellets were mixed in 0.7 ml of chloroform:methanol (2:1) in BeatBUG^®^ vials by BeatBUG^®^ homogenizer (2 cycles per 10 s at 400 rpm). After centrifugation in a Micro 200 R Hettich centrifuge set to 4 °C for 5 min at 10,000 rpm, the collected supernatant was transferred to a 2-ml Eppendorf tube and the homogenization was repeated. Then, 0.4 ml of deionized water was added to the coupled supernatants, vortexed vigorously, and centrifuged (same settings). After centrifugation, the lower lipid phase was transferred into pre-weighted tubes and subsequently evaporated under nitrogen. The final determination of the lipid content was carried out gravimetrically (Mettler Toledo XP6 Excellence Plus XP Micro Balance, 6.1 g x 1Ug, Greifensee, Switzerland).

Fatty acid analysis was performed at the Laboratory of Nutrition, Faculty of Fisheries and Protection of Waters, University of South Bohemia in České Budějovice (Czech Republic) by routinely used gas chromatography with flame ionization detector system^58^. The measurement of fatty acid methyl esters (FAME) profile within extracted lipid from the crayfish whole-body homogenate was done following established protocols^53^. The methylation of lipids was induced with a boron trifluoride-methanol complex solution and NaOH, as described by Appelqvist^59^. FAME 23:0 was used as an internal standard. FA composition was analyzed by gas chromatography (Trace Ultra FID; Thermo Scientific, Milan, Italy) using a BPX-70 50 m fused silica capillary column (id. 0.22 mm, 0.25 μm film thickness, SGE, USA). The temperature gradient was initiated at 70 °C and was held constant for 0.5 min. Then, the temperature was raised by 30 °C per min until it reached 150 °C. After that, the temperature was raised to 220 °C at a rate of 1.5 °C per min, where it was maintained for 11 min. The complete analysis took 60 min. The temperature of the PVT injector was 170 °C and the detector 260 °C. Peaks were identified and quantified in Thermo Xcalibur 3.0.63 (Thermo Fisher Scientific Inc.) by comparing sample retention times and peak areas at seven levels (15 µg/ml – 1000 µg/ml) of the standard mixture Supleco 37 Component FAME mix (Sigma-Aldrich). All samples were analyzed in duplicate.

In terms of the samples, three wild-caught mature marbled crayfish (pooled into one whole body homogenate) served as a wild (natural) control for the fatty acids body profile. At the end of the experiment, the feed and the crayfish fed on the respective diets were sampled. To reflect the body fatty acids composition under each diet (fed for 100 days), 12 marbled crayfish in all (mixing all size groups) per group were pooled (4 marbled crayfish × 3 replicate tanks). The crayfish body lipid content is quite low (0.8–1.3% of wet weight), so pooling was necessary (a) to prepare a representative sample of finely ground whole-body homogenate^53^ that was sufficient for lipid extraction and analysis in ‘duplicate’; and (b) to cover the nuances of social hierarchy (size heterogeneity) on feed intake, which can lead to heterogeneity in body lipid reserves and FA composition. The pooling approach weakened the statistical power but gave the most representative picture of the feed-to-body mass fatty acid flows in the crayfish stock in each experimental feed group. The mixed-size group sampling negated (to some extent) the effect of the cannibalism-assisted fatty acids increase in the bodies of a few larger crayfish; the dilution effect was due to the presence of smaller pellet-eating crayfish in the pool.

### Statistical analysis

To find a link with sexual maturity, special emphasis was given to the appearance of glair glands in the tested animals in relation to the feed (supply) or biomass (deposit) of polyunsaturated fatty acids (PUFA), particularly the long-chain fatty acids (> C20; above all, the arachidonic acid – ARA, 20:4n-6, eicosapentaenoic acid – EPA, 20:5n-3, and docosahexaenoic acid – DHA, 22:6n-3). The body fatty acids profile was compared with diet and then retrospectively compared with those of the wild adult body. Following the Shapiro–Wilk normality test, either a parametric (one-way ANOVA + Tukey HSD) or non-parametric statistical test (Kruskal Wallis + Dunn-Bonferroni) was performed at α = 0.05^5^. However, a saturation kinetics model of ARA/EPA/DHA accumulation in crayfish biomass (body lipids) concerning the dietary supply (% share in food lipid) was assessed using a power regression (y = ax^b^). For this purpose, the percentage shares of ARA or EPA or DHA in the total fatty acids (lipids) in the diet were taken as independent variables (x). The accumulation factors of ARA or EPA or DHA in crayfish biomass (fed on that diet) were taken as dependent variables (y). The accumulation factor of a given fatty acid was calculated as: [% share of ARA or EPA or DHA in the lipid of crayfish biomass fed a particular diet (numerator) divided by % share of ARA or EPA or DHA in the lipid of that diet (denominator)]. This accumulation factor has been used elsewhere in the assessment of resource-to-biomass conversion of fatty acids in aquatic animals^53,60^.

### Ethical statement

All procedures performed followed national and institutional experimental and ethical standards.

### Supplementary Information

Supplementary Information.

## Data Availability

All the data included in the manuscript and supplementary data are freely available.
